# Quercetin shows anti‐tumor effect in hepatocellular carcinoma LM3 cells by abrogating JAK2/STAT3 signaling pathway

**DOI:** 10.1002/cam4.2388

**Published:** 2019-07-05

**Authors:** Liwei Wu, Jingjing Li, Tong Liu, Sainan Li, Jiao Feng, Qiang Yu, Jie Zhang, Jiaojiao Chen, Yuting Zhou, Jie Ji, Kan Chen, Yuqing Mao, Fan Wang, Weiqi Dai, Xiaoming Fan, Jianye Wu, Chuanyong Guo

**Affiliations:** ^1^ Department of Gastroenterology Shanghai Tenth People's Hospital, Tongji University School of Medicine Shanghai China; ^2^ Department of Gastroenterology Putuo People's Hospital, Tongji University School of Medicine Shanghai China; ^3^ Shanghai Tenth Hospital, School of Clinical Medicine of Nanjing Medical University Shanghai China; ^4^ Department of Gerontology Shanghai General Hospital, Shanghai Jiaotong University School of Medicine Shanghai China; ^5^ Department of Oncology Shanghai General Hospital, Shanghai Jiaotong University School of Medicine Shanghai China; ^6^ Department of Gastroenterology Zhongshan Hospital of Fudan University Shanghai China; ^7^ Shanghai Institute of Liver Diseases, Zhongshan Hospital of Fudan University Shanghai China; ^8^ Department of Gastroenterology Jinshan Hospital of Fudan University, Jinshan Shanghai China

**Keywords:** apoptosis, autophagy, hepatocellular carcinoma, JAK2/STAT3 pathway, metastasis, quercetin

## Abstract

**Objective:**

Hepatocellular carcinima is one of the most common tumors in clinic and also one of the leading causes of death from cancer worldwide. Quercetin shows significant effects on blocking the development of various cancers.

**Methods:**

We used the human hepatocellular carcinoma LM3 and nude mice tumor model to assess the effects of quercetin in hepatocellular carcinoma and clarify its mechanism of action. We collected LM3 cell line treated with different doses of quercetin at different time periods and determined the vital indexes. The liver tissues of mice were collected and used for western boltting (WB), Hematoxylin and Eosin (H&E) and TUNEL staining.

**Results:**

Results indicated that quercetin suppressed the Hepatocellular carcinoma (HCC) growth both in vivo and in vitro. Quercetin could disturb LM3 cells proliferation and cell cycle distribution, thus inducing apoptosis. At the same time, quercetin inhibited LM3 cells migration and invasion and promoted HCC autophagy. These effects at least partly depended on the down‐regulation of the activation of JAK2 and STAT3 by quercetin.

**Conclusion:**

Quercetin inhibited hepatocellular carcinoma progression by modulating cell apoptosis, migration, invasion, and autophagy; and its effects were at least partly related with the JAK2/STAT3 signaling pathway.

## 1 INTRODUCTION

Hepatocellular carcinoma (HCC) is a common malignant tumor with the highest incidence of clinic, and it is also one of the top causes of cancer‐induced deaths worldwide. It is commonly known to show vascular invasion, rapid progression, and poor prognosis. In addition, HCC is a polygenic chronic disease, which involved complex genes and signaling pathways; as a result, there are many obstacles on the way to achieving a successful therapeutic goal. Therefore, searching for more efficient antitumor drugs to treat HCC remains a highly important research area.(Figure S1)

Flavonoids can be widely found in our daily lives, such as fruits, vegetables, tea, coffee, and wine. And they have strong anti‐inflammatory and anti‐oxidant effects^1^. Thus, they can protect organs against damages caused by biological, chemical, and physical factors. Flavonoids also have therapeutic anticancer functions. Epidemiology studies have shown that flavonoids can lower the risk of occurrence of many chronic diseases, such as cancer, caused by poor lifestyle and age.^2^, ^3^ Studies have shown that flavonoids can play its roles through many genes and pathways, for example, nuclear factor kappa B(NF‐kB), phosphatidylinositol3'‐kinase (PI3K), signal transducers and activators of transcription (STAT), and tumor necrosis factor‐related apoptosis‐inducing ligand (TRAIL), to protect human health.^4^, ^5^, ^6^

Quercetin(3,3′,4′,5,7‐Pentahydroflavone,QE) is a typical flavonoid that shows significant effects on blocking the development of breast cancer,^7^ ovarian cancer,^8^ carcinoma of colon and rectum,^9^ gastric cancer,^10^ and hepatic cancer. Our previous studies have shown that quercetin could protect the liver from concanavalin A‐induced acute hepatitis, ischemia reperfusion injury, and fibrosis.^11^, ^12^, ^13^ Others have reported that quercetin had effective anticancer functions in hepatocellular carcinoma^1^, ^6^, ^14^, ^15^ and this function may have a close relationship with the STAT3 pathway.^8^, ^16^, ^17^, ^18^ As a cytokine, the transcription factor STAT3, has been testified that it is closely associated with tumor development. STAT3 can inhibit apoptosis and autophagy,^19^, ^20^, ^21^ and promote migration/invasion^16^, ^22^, ^23^ of primary cancers, such as liver cancer. After activation, STAT3 will translocate into the nucleus and regulate transcription. However, most studies on the relationship among quercetin, HCC and STAT3 signaling were incomplete. The objective of our study is to assess the anticancer effects of quercetin and clarify its mechanism of action.

## 2 MATERIALS AND METHODS

### 2.1 Reagents

Quercetin, AG490, IL‐6 and DMSO were obtained from Sigma Chemical Co. (St. Louis, MO). Fetal bovine serum (FBS), trypsin, and Dulbecco's Modified Eagle's Medium (DMEM) were purchased from Gibco (Thermo Fisher Scientific, Waltham, MA). Anti‐N‐cadherin, anti‐Vimentin, anti‐E‐cadherin, anti‐MMP‐9, and anti‐Cyclin B1 antibodies were purchased from Abcam (Cambridge, UK). Anti‐Bax, anti‐LC3, anti‐Beclin1, anti‐PCNA and anti‐STAT3, anti‐phospho‐STAT3, anti‐total‐JAK2, anti‐phospho‐JAK2 antibodies were obtained from Cell Signaling Technology (Danvers, MA). Antibody to anti‐P62 was obtained from Proteintech (Chicago, IL).

### 2.2 Cell lines and cultures, and morphological analyses

The HCC cell line LM3 was purchased from the Chinese Academy of Sciences Committee Type Culture Collection Cell Bank (Beijing, China). These cell lines were cultured in high glucose DMEM (DMEM‐h) supplemented with 10% FBS, 100 U/mL penicillin, and 100 mg/mL streptomycin in a humidified incubator at 37°C in 5% CO_2_.

For morphology experiments, cells were cultured with indicated treatments for 48 hours. Cell morphology was observed using a phase‐contrast microscope and imaged (400×).

### 2.3 Cell viability

HCC cell line LM3 was plated at a density of 4 × 10^4^ cells/ml in 96‐well plates (100 µL medium per well) with five replicates. HCC cells were treated with QE at doses of 0, 20, 40, 60, 80, 100,120, 140, 160, and 200 µmol/L. After seeding for 24, 48, and 72 hours, cell proliferation and viability were measured using the CCK8 assay (Dojindo Laboratories, Tokyo, Japan) according to the manufacturer's instructions.

### 2.4 Flow cytometry analysis

HCC cells in logarithmic growth phase were seeded into 6‐well plates and exposed to QE (40, 80, and 120 µmol/L) for 48 hours. The cells were digested and washed with phosphate‐buffered saline (PBS) for two times. The pellets were suspended in 1× binding buffer, and incubated with annexin‐V/propidium iodide (PI: BD Biosciences, San Jose, CA) following the standard steps. The samples were examined using flow cytometry (Cytomics FC500; Beckman Coulter, Fullerton, CA).

After similar processing, the cells were fixed with 75% ethanol at −20°C overnight, and then resuspended in PBS containing 20 mg/mL PI and 50 mg/mL RNase A. The cells were then incubated in the dark for 30 minutes at 4°C and the cell cycle was analyzed using flow cytometry.

### 2.5 Colony formation assay

After resuspension, LM3 cells were plated into 6‐well plates and were treated with vehicle or QE (80 and 120 µmol/L). After 14 days, the colonies developed in each well were photographed and counted.

### 2.6 Wound‐healing assay in vitro

The LM3 cells were planted in 6‐well plates, and when the cell fusion reached 80%, we used the tip of a 200 mL pipette to cut the cells' surface and form a wound. After the cells were washed three times, they were treated with DMEM (without FBS) or QE (80 and 120 µmol/L) and incubated for 36 hours to quantify the wound healing process. The distance of the scratch closure was examined at 12, 24, and 36 hours. At least ten microscopic fields were analyzed, and representative figures are shown.

### 2.7 Transwell invasion assays

Experiments were performed using a Transwell chamber (BD Biosciences). Basement Membrane Matrix (Corning, Corning, NY) was placed into the upper chamber. DMEM‐h containing 10% FBS was placed in the lower chamber. 1 × 10^5^ cells (LM3) in serum‐free medium were placed in the upper chamber with different concentrations of QE. After 48 hours, the cells attached to lower membrane surface were stained with 0.05% Crystal Violet.

### 2.8 Confocal microscopy

The cells were seeded onto glass cover slips. After 48 hours, treated and untreated cells were washed with PBS and then immersed in the fixation solution. These fixed cells were incubated with antibodies overnight at 4°C. Immunofluorescence was detected with anti‐rabbit immunoglobulin G (IgG) conjugated with Alexa Fluor 488 (green) and DAPI (blue) (Invitrogen, Carlsbad, CA) was used for nuclear staining. The immunofluorescence was observed using a confocal microscope (Olympus, Tokyo, Japan).

TUNEL staining was performed using an EdUTP TUNEL cell detection kit (Ribobio, Gangzhou, China) according to the manufacturer's instructions, and was observed using a confocal microscope.

### 2.9 Animal experiments

Animal experiments were performed according to the National Institutes of Health Guidelines for the Care and Use of Laboratory Animals and were approved by the Animal Care and Use Committee of Shanghai Tongji University, China.

Ten nude mice were raised in an environment meeting international standards and were monitored daily. LM3 HCC cells were subcutaneously injected into the upper lateral area of mice at the density of 5×10^6^/100μL. After 1 week, the subcutaneous tumor tissue grew to the size of about 1.0 mm^3^. All mice were randomly divided into two groups (five mice in each group) according to whether they received QE intervention or not.

QE was suspended in saline and administered for 21 days by gavage at the dose of 100 mg/kg (200 µL for every model mouse). Tumor volume and mouse weight were measured every 3 days and the mice were anesthetized and euthanized. Tumor tissues were harvested for further analysis.

### 2.10 Hematoxylin and eosin staining

A portion of tumor tissue was fixed with 4% paraformaldehyde and embedded into wax blocks. Sections (5 μm thick) were cut, and then were dewaxed and hydrated. Were stored at room temperature. Hematoxylin and eosin (H&E) was added to stain the nuclear regions and cytoplasm. Histopathological changes were observed under a light microscope.

### 2.11 TUNEL (terminal deoxynucleotide transferase dUTP nick end labeling) assays

Apoptosis of tumor tissues was assessed using the TUNEL assay. Paraffin‐embedded sections (5 µm) were cut and mounted on glass slides. After treatment according to the instructions, sections were incubated with the TUNEL reaction mixture for 1 hour at 37°C. Sections were observed under a light microscope.

### 2.12 Protein extraction and western blotting

Total protein from cells or tissues was isolated using radioimmunoprecipitation assay buffer. The protein obtained from supernatants was quantified according to the standard protocol. Proteins were separated using sodium dodecyl sulfate‐polyacrylamide gel electrophoresis and transferred onto polyvinylidene difluoride or nitrocellulose membranes. After incubation with primary antibodies and secondary antibody, blots were developed by using the Odyssey Two‐color Infrared Laser Imaging System (Li‐Cor, Lincoln, NE). The signal generated by β‐actin was used as an internal control.

### 2.13 RNA extraction and quantitative real‐time (qRT)‐PCR analysis

Total RNA was extracted by using TRIzol reagent according to the standard protocol and first‐strand cDNA was synthesized using the reverse transcription kit (TaKaRa Biotechnology, Dalian, China). The cDNA was used in real‐time PCR reactions to analyze indicators expression. Primers used in the PCR reactions are listed in Table 1. The real‐time PCR experiments were performed according to the protocol of the real‐time PCR kit (Takara, Otsu, Shiga, Japan). The expression of those genes was calculated using the 2^(‐ΔΔC(T))^ method.

**Table 1:** Nucleotide sequences of primers used for qRT‐PCR

Gene	Primer Sequence(5'‐3')	
	Forward	Reverse
PCNA	GCTGACATCGGACACTTA	CTCAGGTACAAACTTGGTG
Bax	AAGAAGCTGAGCGAGTGT	GGAGGAAGTCCAATGTC
P62	GCACCCCAATGTGATCTGC	CGCTACACAAGTCGTAGTCTGG
LC3	AACATGAGCGAGTTGGTCAAG	GCTCGTAGATGTCCGCGAT
MMP9	TGTACCGCTATGGTTACACTCG	GGCAGGGACAGTTGCTTCT
vimentin	GACGCCATCAACACCGAGTT	CTTTGTCGTTGGTTAGCTGGT
E‐cadherin	CGAGAGCTACACGTTCACGG	GGGTGTCGAGGGAAAAATAGG
β‐actin	CTGGAACGGTGAAGGTGACA	AAGGGACTTCCTGTAACAATGCA

### 2.14 Statistical analysis

All the experiments were conducted three times and were analyzed using Graph Pad Prism 5.0 software. Comparisons among multiple groups were conducted using one‐way ANOVA with the Student's *t* test to compare between two groups. A value of *P* < 0.05 was considered statistically significant.

## 3 RESULTS

### 3.1 QE suppressed LM3 cell viability and induced LM3 cell apoptosis

**Figure 1:**
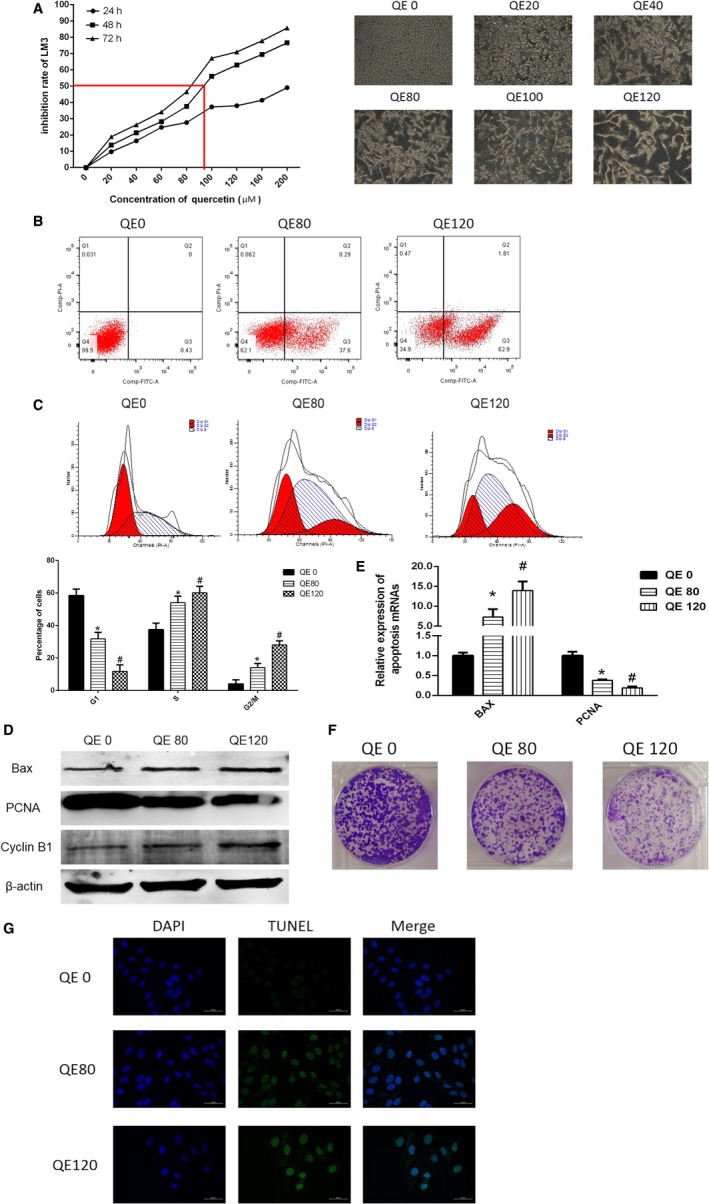
QE inhibited LM3 proliferation and cycle distribution, and induced apoptosis. A, LM3 cells were treated with QE (0‐200 μmol/L) for 24, 48 and 72 h. The CCK8 kit was used to monitor cell proliferation and morphological changes in LM3 cells for 48 h (magnification 400×). B, Apoptosis of LM3 cells was determined using flow cytometry. C, Cell cycle distribution of LM3 cells was determined using flow cytometry. The data are expressed as mean ± SD (*P < 0.05 for QE80 vs QE0, and ^#^ *P* < 0.05 for QE120 vs QE0). D, The protein expression of PCNA, Bax and CyclinB1 were measured using western blot. E, The mRNA expression of PCNA and Bax were measured using qRT‐PCR. The data are expressed as mean ± SD (*P < 0.05 for QE80 vs QE0, and ^#^ *P* < 0.05 for QE120 vs QE0). F, Colony formation of LM3. G, TUNEL staining of LM3 cells was observed after treatment of QE for 48 h (magnification 400×)

The HCC cell line, LM3, was intervened by QE (0, 20, 40, 60, 80, 100, 120,160, and 200 μmol/L) for 24, 48, and 72 hours, whose survivability was measured using CCK8 kits. Cell growth curves were constructed on the basis of data obtained. The analyzed results showed that QE played an inhibition role of the viability of LM3 cells varing with dose and time. We calculated the half maximal inhibitory concentration at 48 hours, which is shown in Figure 1A. QE also exhibited typical morphological changes in LM3 cells. We selected effective QE concentrations (80 and 120 μmol/L) for treatment of LM3 cells for the following experiments.

To assess the extent of apoptosis induced in LM3 cells after QE treatment, flow cytometry, western blotting, qRT‐PCR, colony formation assays, and immunofluorescence were performed. Apoptotic cell death was divided into early stage apoptotic cell death and late stage apoptotic cell death, which are marked as annexin‐V+/PI− and annexin‐V+/PI+. And cell death caused by apoptosis was quantified by the percentage of them. The results demonstrated a growth in the proportion of early stage apoptotic cells in a concentration‐dependent manner after treatment with QE (Figure 1B). We collected protein and RNA from cells treated with QE (0, 80 and 120 μmol/L for 48 hours), and determined the protein and gene expression levels. PCNA is an index associated with DNA synthesis, and can reflect the proliferation of cells. Bax is a classical index for promoting apoptosis. Figure 1D,E exhibit the blots and data; they show that QE reduced the expression of PCNA, and increased the expression of Bax. The results in Figure 1F show that QE suppressed the formation of colonies. In addition, the cleaved DNA in apoptotic cells combined with the TUNEL reagent and showed bright green fluorescence. Figure 1G shows that QE increased the fluorescence intensity of TUNEL. After these cancer cells were treated with QE at doses of 0, 80, and 120 μmol/L for 48 hours, we performed PI staining to measure the distribution of cell cycle. The results reveal that QE treatment induced cells were arrested in the S and G2/M phases, and the number of G0/G1 phase cells was reduced (Figure 1C). Moreover, the protein expression level of cyclin B1, a cell cycle‐related protein, was decreased by treatment with QE as shown by western blotting (Figure 1D).So, we concluded that the inhibition effect of QE in cell proliferation may have a relationship with the cell cycle arrest.

### 3.2 QE inhibited LM3 cell migration and invasion

**Figure 2:**
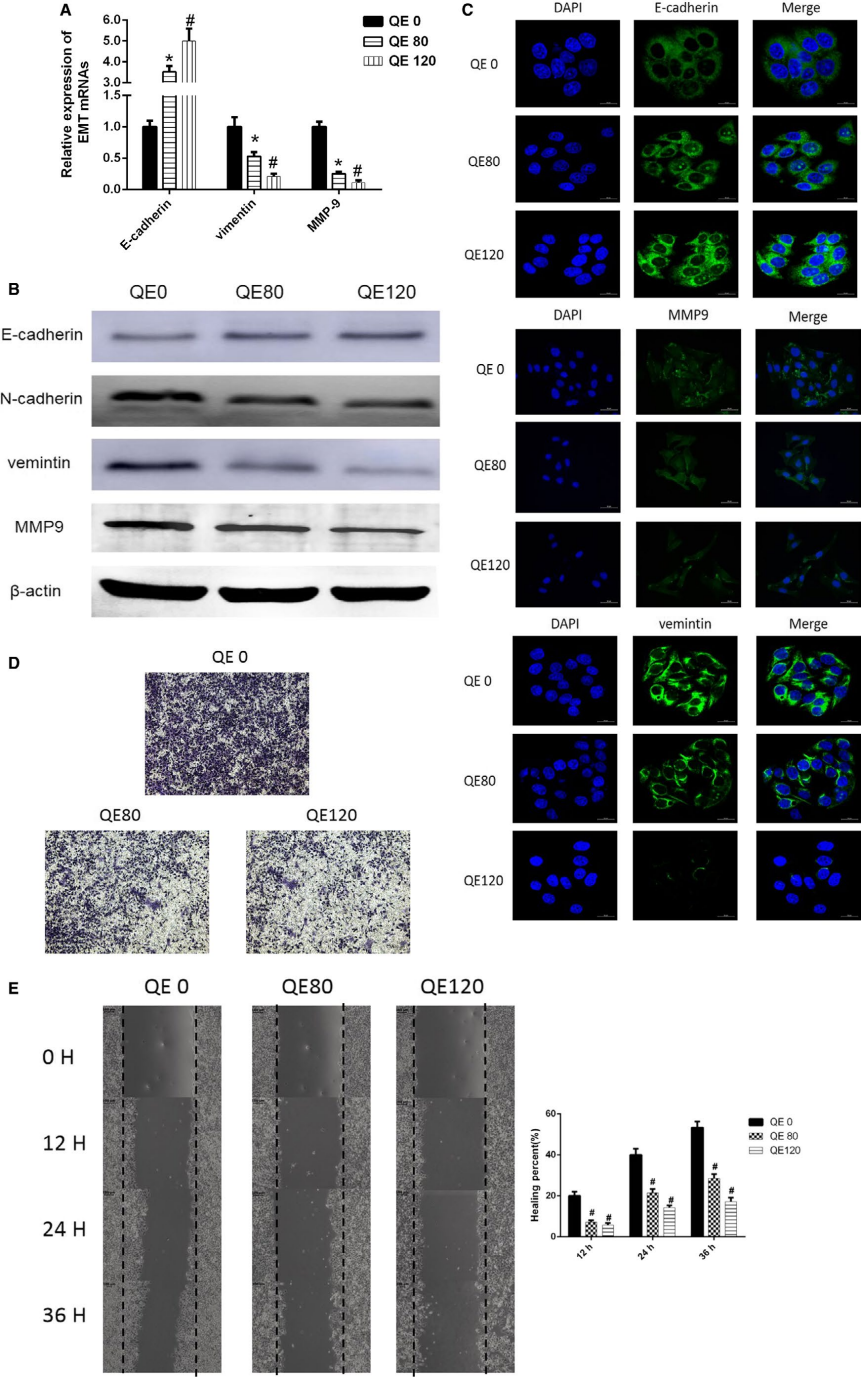
QE inhibits LM3 cells migration and invasion. A, The mRNA expression of E‐cadherin, Vimentin, and MMP9 were measured using qRT‐PCR. The data are expressed as mean ± SD (*P < 0.05 for QE80 vs QE0, and ^#^ *P* < 0.05 for QE120 vs QE0). B, The protein expression of N‐cadherin, E‐cadherin, Vimentin, and MMP9 were measured using western blot. C, The expression of E‐cadherin, Vimentin, and MMP9 in LM3 cells were examined using immunofluorescence staining (magnification 400×). D, Representative images of transwell invasion assays for the inhibitory effect of QE on the invasion ability of LM3 cells. E, Wound healing assay for demonstrating the inhibitory effect of QE on the migration of LM3 cells at 0, 12, 24 and 36 h following wounding. The data are expressed as mean ± SD (^#^ *P* < 0.05 for QE80, QE120 vs QE0)

We then treated LM3 cells with QE at concentrations of 0, 80, and 120 µmol/L for 48 hours, and detected the mRNA levels of important epithelial‐mesenchymal transition (EMT) biomarkers, E‐cadherin, vimentin, and matrix metallopeptidase 9 (MMP9). The results showed that QE escalated mRNA expression levels of E‐cadherin, and reduced mRNA expression of vimentin and MMP9 with QE concentrations increasing (Figure 2A). Furthermore, we performed western blots to detect the protein levels of N‐cadherin, E‐cadherin, vimentin and MMP9 (Figure 2B). Immunofluorescence assays were performed to set out the protein levels of E‐cadherin, vimentin and MMP9, and we found that the results were in line with the trend obtained in previous experiments (Figure 2C). From what has been discussed above, our results revealed that QE reversed the EMT process of LM3 cells. Then, we performed transwell invasion (Figure 2D) and wound healing (Figure 2E) assays to testify the inhibition effect of QE on HCC cells' migration and invasion. The observed results expressed that invasion and migration were significantly restrained by treatment with QE.

### 3.3 QE promoted HCC autophagy

**Figure 3:**
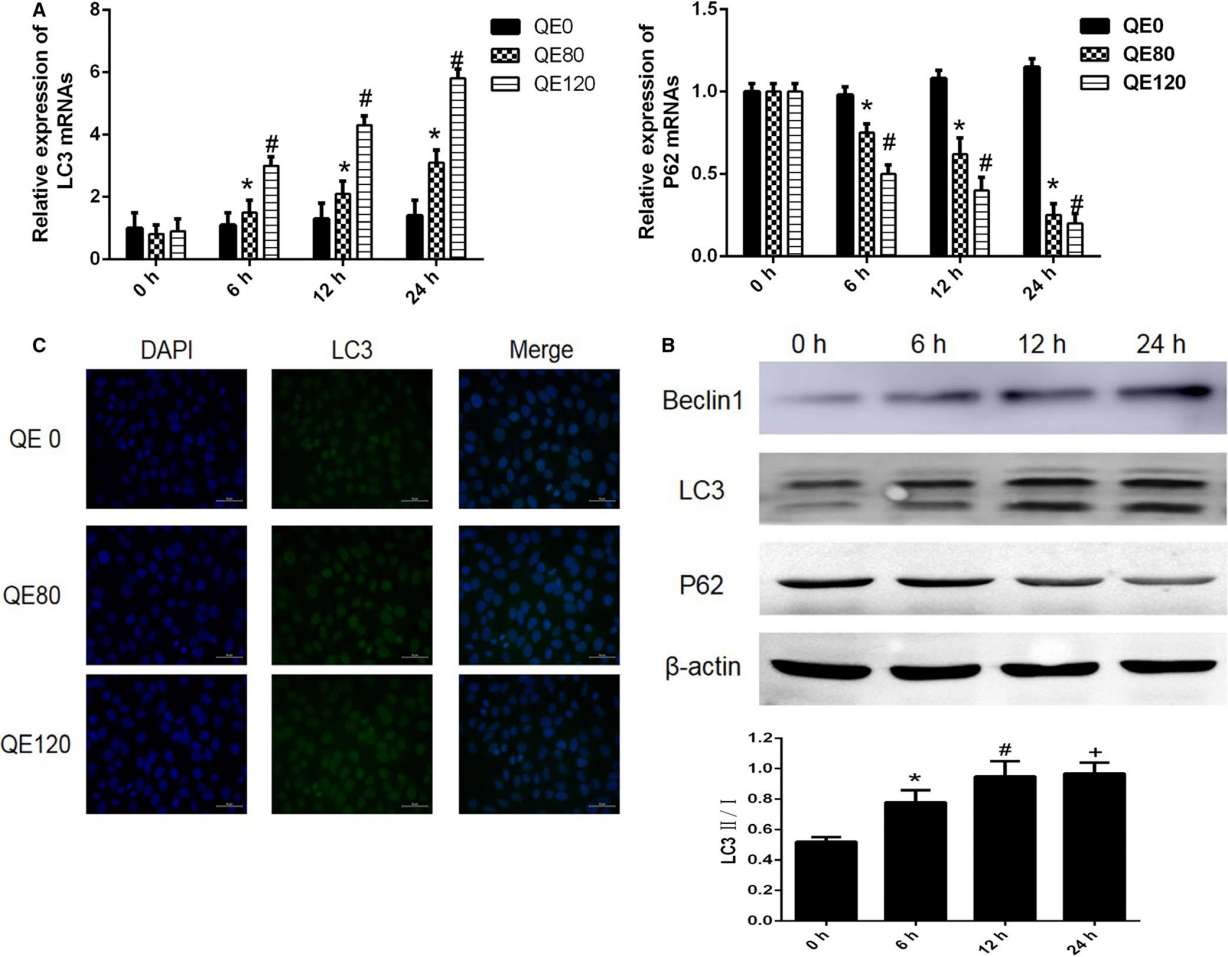
QE promotes HCC autophagy. A, The mRNA expression of LC3 and P62 were measured using qRT‐PCR. The data are expressed as mean ± SD (*P < 0.05 for QE80 vs QE0, ^#^ *P* < 0.05 for QE120 vs QE0). B, The protein expression of Beclin1, LC3 and P62 were measured using western blot and the ratio of LC3ǁ/ǀ is shown. C, The expression of LC3 in LM3 cells was examined using immunofluorescence staining (magnification 400×)

Autophagy is the process of cell phagocytosis of cytoplasmic proteins or organelles and degradation, to meet the requirement for renewal of some organelles and the metabolism of cells. We explored whether QE could activate autophagy in HCC cells. Beclin‐1, LC3 and P62 are typical biomarkers for autophagy. As shown in Figure 3A, significantly up‐regulated expression of LC3, and down‐regulated expression of P62 were observed after QE treatment in a dose and time‐dependent manner. Furthermore, we treated cells with QE (80 μmol/L) and analyzed the proteins at 0, 6,12, and 24 hours after QE treatment using western blot analyses. The results were in accordance with the qRT‐PCR results, which are shown in Figure 3B. The results of immunofluorescence assays showed the up‐regulation of LC3 by QE treatment (Figure 3C). Taken together, QE induced autophagy in HCC cells.

### 3.4 QE inhibited the activation of JAK2/STAT3 pathway

**Figure 4:**
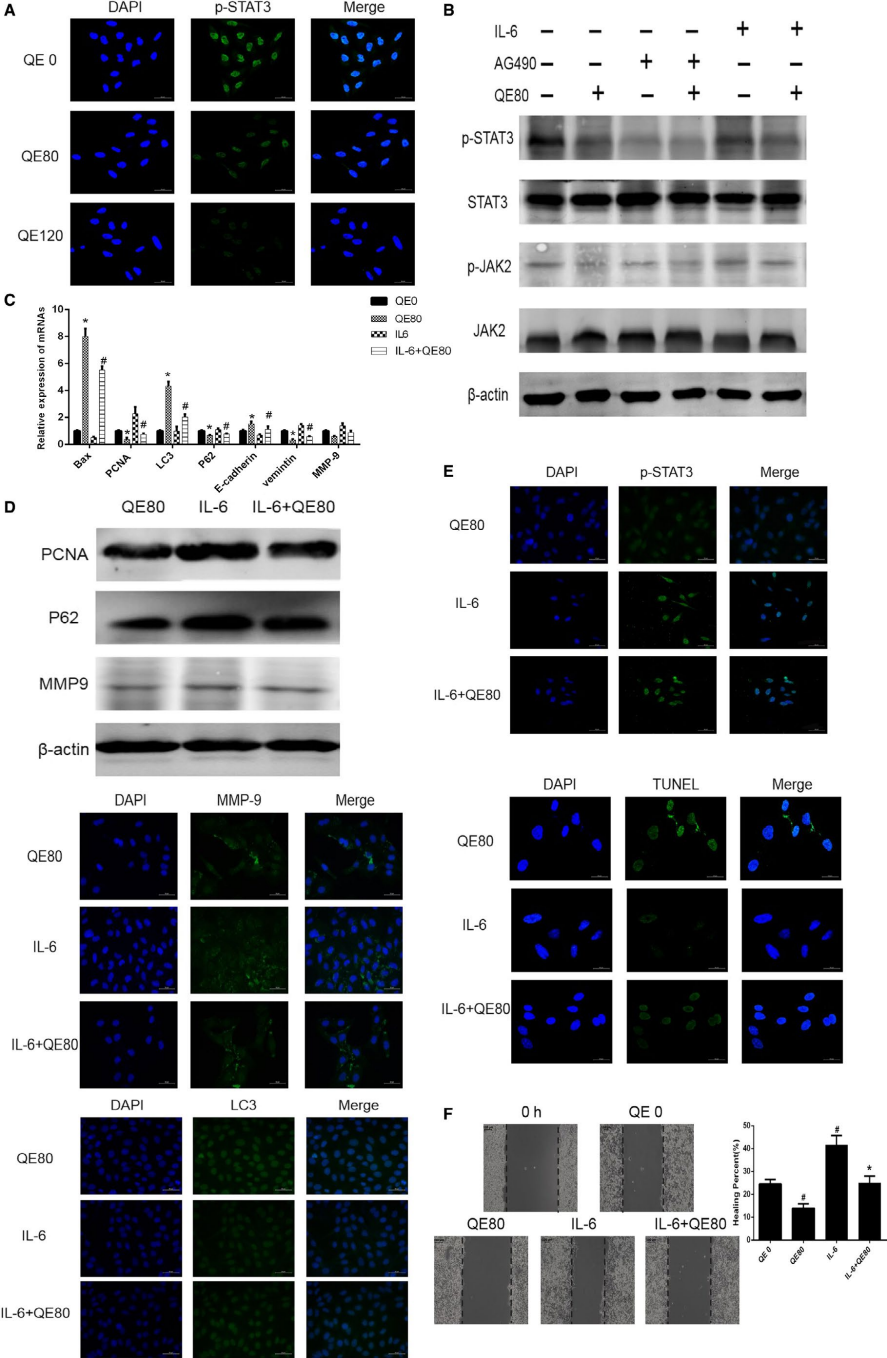
QE inhibited STAT3 signaling pathway. A, The expression of p‐STAT3 in LM3 cells was examined using immunofluorescence staining (magnification 400×). B, The protein expression of STAT3 pathway was measured using western blot. C, The mRNA expression of Bax, PCNA, LC3,P62,vimentin and MMP9 were measured using qRT‐PCR. The data are expressed as mean ± SD (*P < 0.05 for IL‐6 vs QE80, ^#^ *P* < 0.05 for IL‐6 vs IL‐6 + QE80). D, The protein expression of PCNA, P62 and MMP9 were measured using western blot. E, The expression of p‐STAT3, MMP9, TUNEL and LC3 in LM3 cells were examined using immunofluorescence staining (magnification 400×). F, Wound healing assay for demonstrating the effect of QE and IL‐6 on the migration of LM3 cells at 12 h following wounding. The data are expressed as mean ± SD (^#^ *P* < 0.05 for QE80,IL‐6 vs QE0, *P < 0.05 for IL‐6 + QE80 vs IL‐6)

QE is able to affect various signaling pathways, for example, the STAT3 pathway. To make sure whether QE could modulate the activation of the STAT3 pathway, we detected the expression of P‐STAT3 in LM3 cells by immunofluorescence staining. The results shown in Figure 4A suggested that QE reduced the expression of p‐STAT3, and therefore, we hypothesized that the antitumor effect of QE was related with the STAT3 pathway. AG490 and IL‐6 were confirmed that they could take an inhibitor or promoter role in JAK2/STAT3 signaling in various cells. And then, we used AG490(50μM), IL‐6(100ng/ml) and QE(80μM) to treat LM3 cells ,and collected their protein. The changed expression of STAT3 and JAK2 was as shown in Figure 4B. We found that there was no obvious change of the STAT3 and JAK2 expression in cells after treatment with AG490, IL‐6 or QE. However, the expression changes of phosphorylated STAT3 and JAK2 induced by AG490 could be enhanced by QE, and QE could reverse the changed expression caused by IL‐6. In conclusion, QE could inhibit the activation of the JAK2/STAT3 pathway effectively.

Then, we used qRT‐PCR, WB, wound healing and immunofluorescence staining to confirm whether QE could reverse STAT3 signaling induced apoptosis, metastasis and autophagy. The results of above experiments were exhibited in Figure 4C‐F, which testified our hypothesis. QE significantly showed opposite effect of IL‐6, and could weaken its influences. The changes caused by IL‐6 on PCNA, P62 and MMP‐9 protein expression were changed by QE, and images of immunofluorescence staining show the same trend. Besides, the speed of wound healing was expedited by IL‐6 and retarded by QE. Briefly speaking, QE could reverse the changes induced by STAT3 pathway, and that is to say, the antitumor effect of QE at least partly depended on the STAT3 signaling pathway.

### 3.5 QE inhibited LM3 cell tumor growth in an animal model

**Figure 5:**
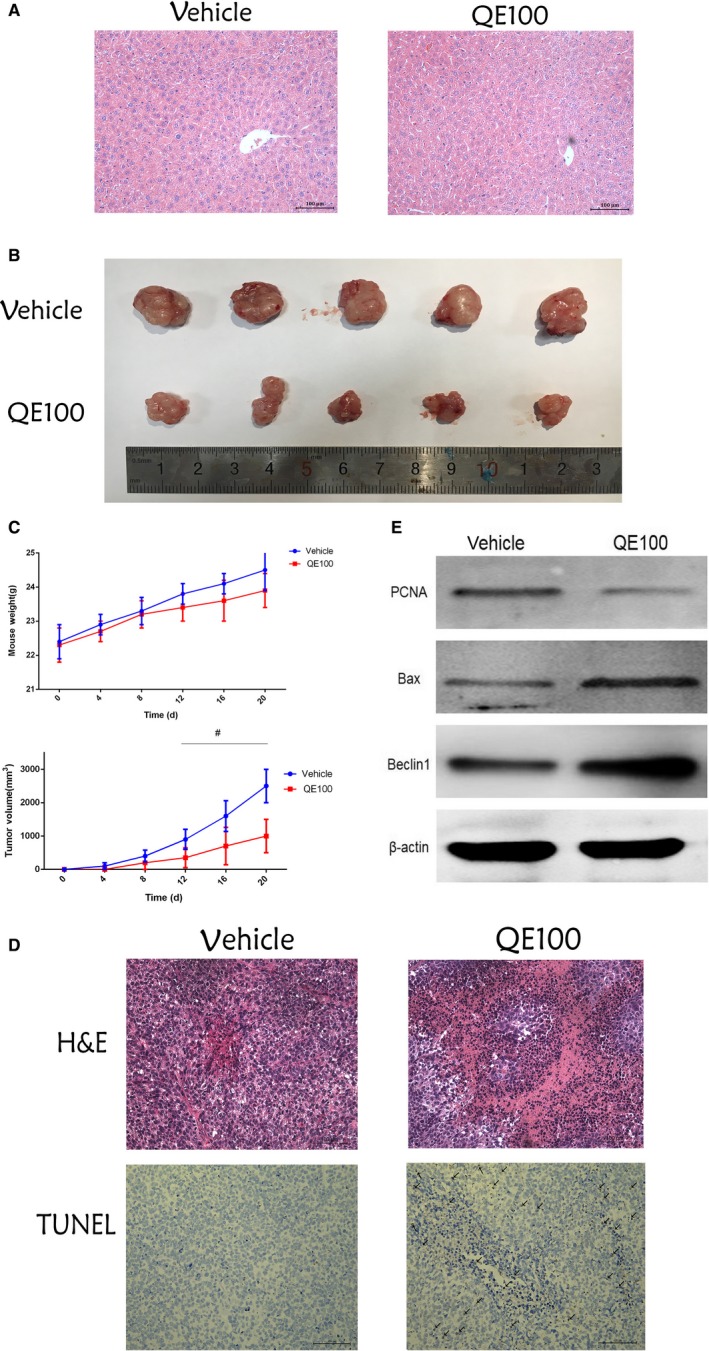
QE inhibited tumors growth in vivo. A, HE staining of mice (magnification 200×). B, Gross observation of HCC‐LM3 cell xenograft tumors in nude mice. C, The changes in body weights and tumor volume were recorded at the time points indicated. The data are expressed as mean ± SD (^#^ *P* < 0.05 for QE100 vs vehicle). D, HE and TUNEL staining of tumors show the level of necrosis and apoptosis (magnification 200×). E, The protein expression of PCNA and Bax were measured using western blot

Figure 5A shows that QE has no obvious adverse effect on the liver. Several nude mice were given an injection of LM3 cells subcutaneously, and then received saline or QE (100 mg/kg) treatment to evaluate the in vivo effect of QE for HCC progression. QE treatment significantly downsized tumor volume compared to the control group by approximately 70% (Figure 5B), and mouse weights and tumor volume were reduced with time (Figure 5C). H&E staining of tumor tissues showed obvious necrosis in the QE‐treated group. Section staining of tumor tissues analyses showed more TUNEL‐positive cells in the QE group, while being fewer in the vehicle group (Figure 5D). Besides, to clarify the proapoptosis effect of QE in cancer tissues, we measured the expression of PCNA and Bax using western blot. The expression of PCNA was effectively reduced by QE treatment, and Bax levels were increased. Also, the expression of Beclin‐1 was changed in line with in vitro experiments. Together, these results suggested that QE inhibited tumor growth in vivo. (Figure S1)

## 4 DISCUSSION

Malignant tumors are a threat to human health. In China, partly due to the high incidence of hepatitis, hepatic cancer is one of the most common malignant tumors. Current research on anticancer therapy has focused on agents with availability and low toxicity. At this time, Chinese traditional medicine has been studied to identify additional treatments.

Chinese traditional medicines have been studied in various cancers for their preventive and therapeutic properties.^15^ Flavonoids, polyphenolic plant secondary metabolites, have been confirmed having effective antioxidant, antiinflammation, and antiproliferative biological activities, which are beneficial for human health.^24^ Among the flavonoids, QE is a widely studied compound in cancers such as lung and ovarian cancer.^25^, ^26^, ^27^, ^28^ Our previous studies have shown that QE protected the liver against injury, with no obvious harm to the body.

To confirm the anticancer effect of QE, we explored its influence in the following three aspects: apoptosis, metastasis, and autophagy. The ability of tumor cells to proliferate and survive is an important factor in tumorigenesis, where cells with mutations and DNA damage can continue to grow.^29^At present, medical scientists believe that inhibiting cell proliferation and inducing apoptosis are the main entry points of cancer treatment. We used the CCK8 cell viability assay kit to assess the cell viability of LM3, which were treated with QE (20–200 μmol/L), for 24, 48, and 72 hours. The results showed that QE caused time‐ and dose‐dependent inhibition of HCC proliferation. We also used cytometry, western blots, qRT‐PCR, colony formation assays, and immunofluorescence to study the levels of proliferation and apoptosis of LM3. QE has been confirmed to have an antiproliferative effect and to induce cell cycle arrest in many types of cancer cells.^14^ Because PCNA is related to the synthesis of DNA, it is used as an important index of cell proliferation. The expression levels of PCNA supported that tumor cell proliferation was reduced by QE treatment. Apoptotic tolerance is also considered to be a major factor affecting tumor growth and drug resistance.^30^ From the data and pictures we obtained by using flow cytometry and TUNEL assays, the increased proportion of apoptosis and their changes in morphology coincided with the increased expression of PCNA and Bax caused by QE treatment. The above results indicated that QE reduced the proliferation capacity of HCC and increased the incidence of apoptosis. In agreement with previous studies, our results reported a QE‐induced cycle arrest in HCC cells, which was associated with regulation of CyclinB1 expression.^31^Cyclin B1 is a cell cycle‐related protein, which is expressed in S and G2/M phases, especially G2/M. This told us that the effect of QE in inducing apoptosis may depend on its regulation of cell cycle.

Many in vivo and in vitro experiments have proved the positive role of QE in HCC. HepG2, Huh7, SMMC‐7721 and LM3 are typical hepatocellular carcinoma cell lines, and there are many relative reports of HepG2 and Huh7. QE could inhibit HCC proliferation and induce cell cycle arrest^32^, ^33^ by regulating the expression and function of the p53,^34^SP1,^35^PI3K/PKC^36^ and MEK/ERK^37^ pathways. Besides, some researchers showed that QE could reverse multidrug resistance via FZD7/β‐catenin.^38^ Furthermore, QE could combine with nickel,^39^ cisplatin^40^ and sorafenib^41^ and demonstrate a great role in suppressing growth and inducing apoptosis in HCC. Also, several animal experiments were demonstrated to exhibit the protective effect of QE from liver cancer.^1^, ^42^, ^43^ Evidences suggested that its function was related to ROS^44^, ^45^ and autophagy.^42^ At the same time, our study, which was more comprehensive, was operated in LM3 cell line and contained both in vivo and in vitro experiments. Our results showed that the positive effect of QE in HCC was linked with proliferation, apoptosis, autophagy, cell cycle arrest, migration and invasion. The part of autophagy, migration and invasion was seldom mentioned in papers for QE in HCC.

Clinical studies and animal experiments have shown that invasion and migration are two important pathological processes of cancer cells, which are closely associated with cancer mortality, particularly for HCC.^46^, ^47^ Studies have reported that QE influenced the process of invasion and migration. QE was shown to inhibit nickel‐induced human lung cancer cells metastasis by down‐regulation of Toll‐like receptor (TLR)4/NF‐κB signaling.^48^ The results of Liu et al^49^ revealed the proliferation and migration inhibition effects of QE on human glioblastoma. Another study showed that QE could modulate EMT markers and led to EMT transition in negative breast cancer.^50^ Another study clarified the function of QE in inhibiting hepatocyte growth factor‐stimulated migration and invasion.^51^ Erdogan et al^23^ reported QE suppressed prostate cancer stem cells survival and migration. QE could also inhibit migration of medulloblastoma cells,^52^ teratocarcinoma cells,^53^ and breast cancer cells.^54^ Therefore, we evaluated the role of QE on invasion and migration in LM3 cells. Using morphological observations, western blotting, qRT‐PCR, and immunofluorescence analyses, QE was shown to reverse the phenomenon of invasion and migration. QE increased E‐cadherin expression, an epithelial marker, but decreased N‐cadherin and vimentin, mesenchymal markers, at the gene expression and protein levels. These facts suggested that QE inhibited the process of invasion and migration. Most MMPs have been implicated in the tumorigenesis of various human malignancies.^55^ Among these proteases, MMP9 has been reported to be significant in the occurrence of human cancer invasion and metastasis. As evidenced by studies, inhibiting the expression of MMP9 suppressed the metastasis in cancer progression.^22^, ^56^ Our results showed that MMP9 was down‐regulated by QE.

The relationship between the generation rate of autophagy and the progress of tumorigenesis has been studied diffusely, but whether the role of autophagy is tumorigenic or antitumor is unclear.^57^ Some studies have reported that autophagy deficiency is in connection with the clinicopathological properties and adverse outcome of HCC, and that means that autophagy can help suppress the development and progression of tumors.^58^ Thus, stimulating autophagy in tumor cells may be one of the effective means to treat hepatocellular carcinoma.^59^, ^60^ Our results supported this hypothesis. To monitor the level of autophagy, we measured the expression levels of LC3 and p62 at 0, 6, 12, and 24 hours after QE treatment. Our data showed that QE induced autophagic cell death, which contributed to QE cytotoxicity in HCC cells. In addition, these findings supported that autophagy played a tumor‐suppressive role in liver cancer cells treated with QE.

**Figure 6:**
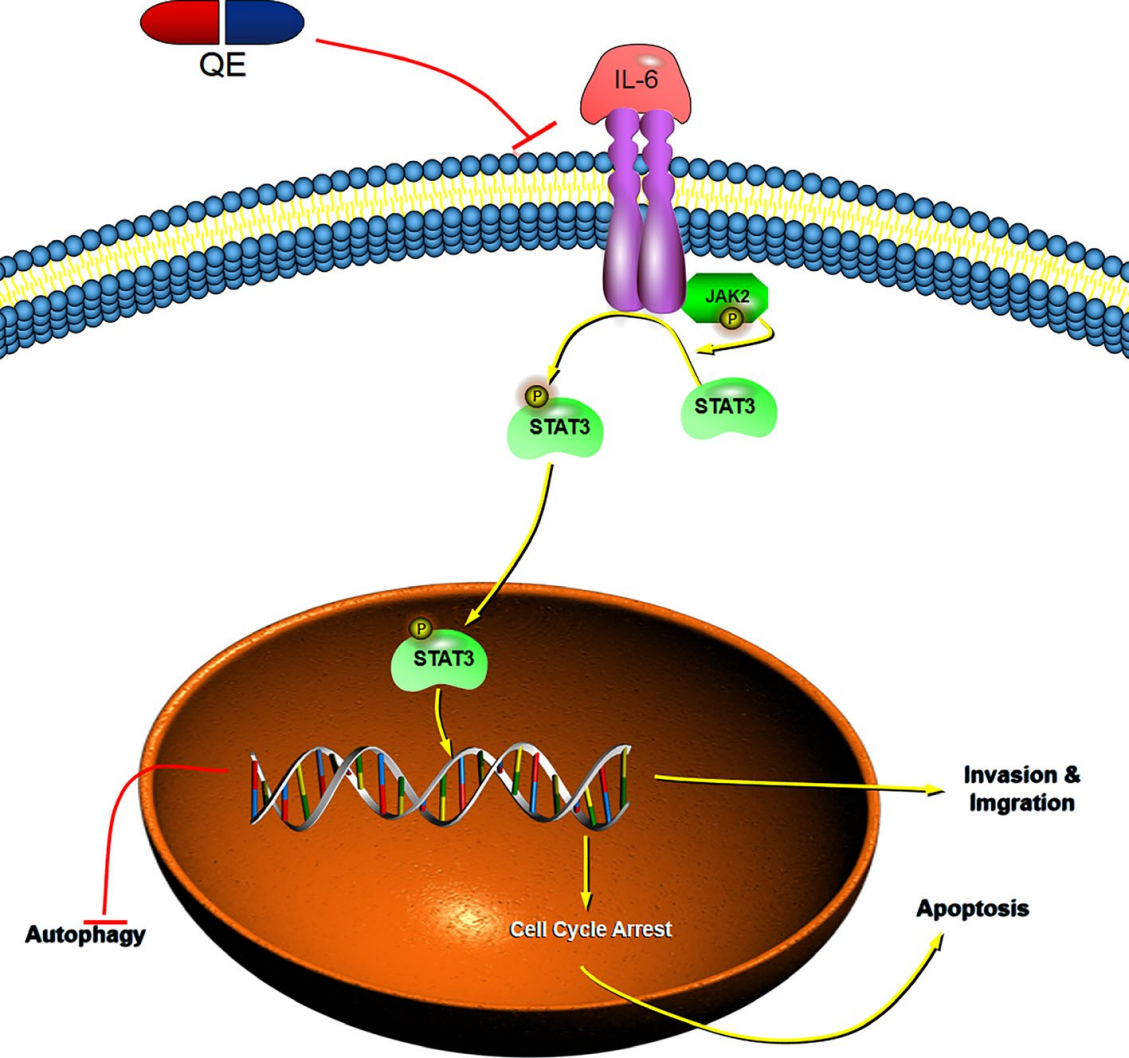
The underlying mechanism of the antitumor effect of QE

Summing up, we have demonstrated that QE inhibited tumor progression by apoptosis, metastasis, and autophagy. Studies have reported that many signalling pathways and transcription factors are involved in tumor progression. Among them, the JAK2/STAT3 signaling pathway plays multiple roles. STAT family has seven members, STAT1, STAT2, STAT3, STAT4, STAT5a, STAT5b, and STAT6,^61^ and STAT3 has been verified for its tumor promotor effect because activation of STAT3 could promote not only tumor cell proliferation and migration, but also angiogenesis and immunosuppression.^21^, ^62^, ^63^ There are some labs tesified the action of STAT3 in liver cancers,^64^, ^65^and the relationship among QE, STAT3 and HCC has been showed by several teams. In various organic damages, the protective function of QE has been proved to be closely related with JAK2 and STAT3 signaling.^66^, ^67^, ^68^, ^69^, ^70^, ^71^AG490 is an inhibitor of JAK2/STAT3, and IL‐6 is a recognized STAT3 activator. The changes, which happened in HCC cells after AG490, IL‐6 or QE treatment, indicated that QE could inhibit the activation of the JAK2/STAT3 pathway. The ability of QE to induce apoptosis and autophagy by blocking STAT3 pathway has been clarified in different cancers, including ovarian cancer,^8^breast cancer,^72^glioblastoma^18^ and liver cancer.^20^It is also testified that the effect of QE is against the biological activity of migration and invasion by inhibiting STAT3.^16^, ^18^ At the same time, our results showed that QE could remit the changes brought by IL‐6.Thus, we confirmed that QE could reverse the changes caused by STAT3 activation (Figure 6). In accordance with expectations, we came to a conclusion, that is, the antitumor effect of QE was at least depending on its abrogation of the JAK2/STAT3 pathway.

Finally, to make sure the practical value of our research, we used a nude mouse model with tumor grafts as an in vivo model. The results showed that tumor growth was significantly delayed, and tumor tissues of the treatment group appeared to have necrosis. We examined the expression of PCNA and Bax, which is effectively reversed by QE treatment. These data suggested that QE slowed hepatic tumor growth in vivo. Taken together, QE inhibited the progression of liver cancer, at least partially, by inhibiting the activation of JAK2/STAT3 signaling pathways. However, whether there are other signals involved in this process needs to be further studied.

## 5 CONCLUSIONS

Quercetin could suppress HCC proliferation and induce apoptosis. Quercetin inhibited LM3 cells metastasis by regulating expression of N‐cadherin, E‐cadherin, vimentin, and MMP9. Also, it promoted HCC autophagy. These effects were related with the JAK2/STAT3 signaling pathway. Our research provided an important and complete evidence for the antitumor function of quercetin. Quercetin has provided a potential therapy for patients with liver cancers.

## CONFLICTS OF INTEREST

The authors declare no conflict of interest.

## Data Availability

The datasets generated during and/or analyzed during the current study are available from the corresponding author on reasonable request.
