# Supplementary figures and images for: Quercetin shows anti‐tumor effect in hepatocellular carcinoma LM3 cells by abrogating JAK2/STAT3 signaling pathway
Source: Cancer Med. 2019 Jul 5;8(10):4806–20. doi: 10.1002/cam4.2388 (PMC6712453; doi:10.1002/cam4.2388)

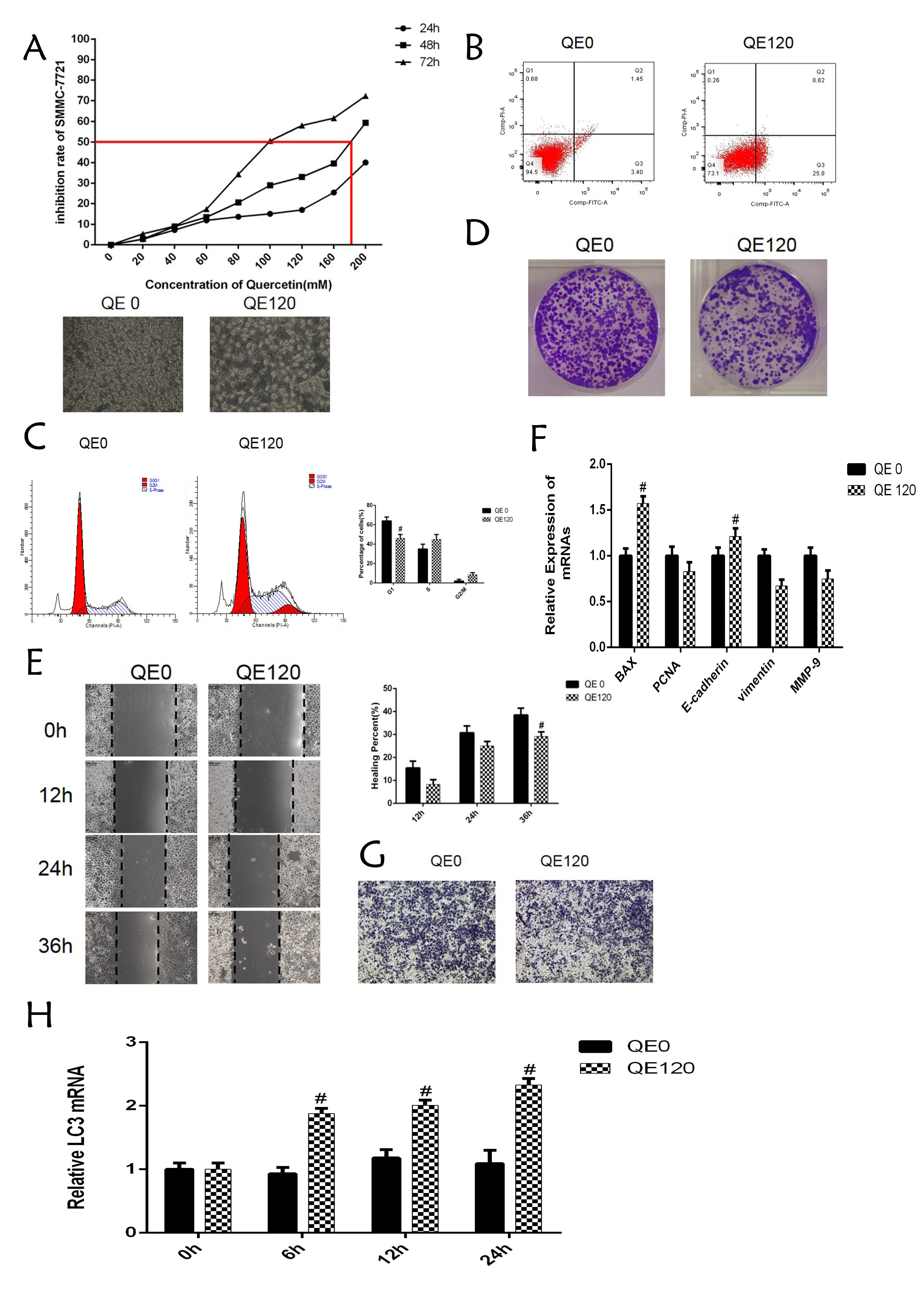

Supplement: Supplementary file 1 [file CAM4-8-4806-s001.tif]
