# Supplementary material for: Quercetin shows anti‐tumor effect in hepatocellular carcinoma LM3 cells by abrogating JAK2/STAT3 signaling pathway
Source: Cancer Med. 2019 Jul 5;8(10):4806–20. doi: 10.1002/cam4.2388 (PMC6712453; doi:10.1002/cam4.2388)
Supplement: Supplementary file 2 [file CAM4-8-4806-s002.docx]

**Supplementary Figure.1 QE showed anti-tumor effect in SMMC-7721.**


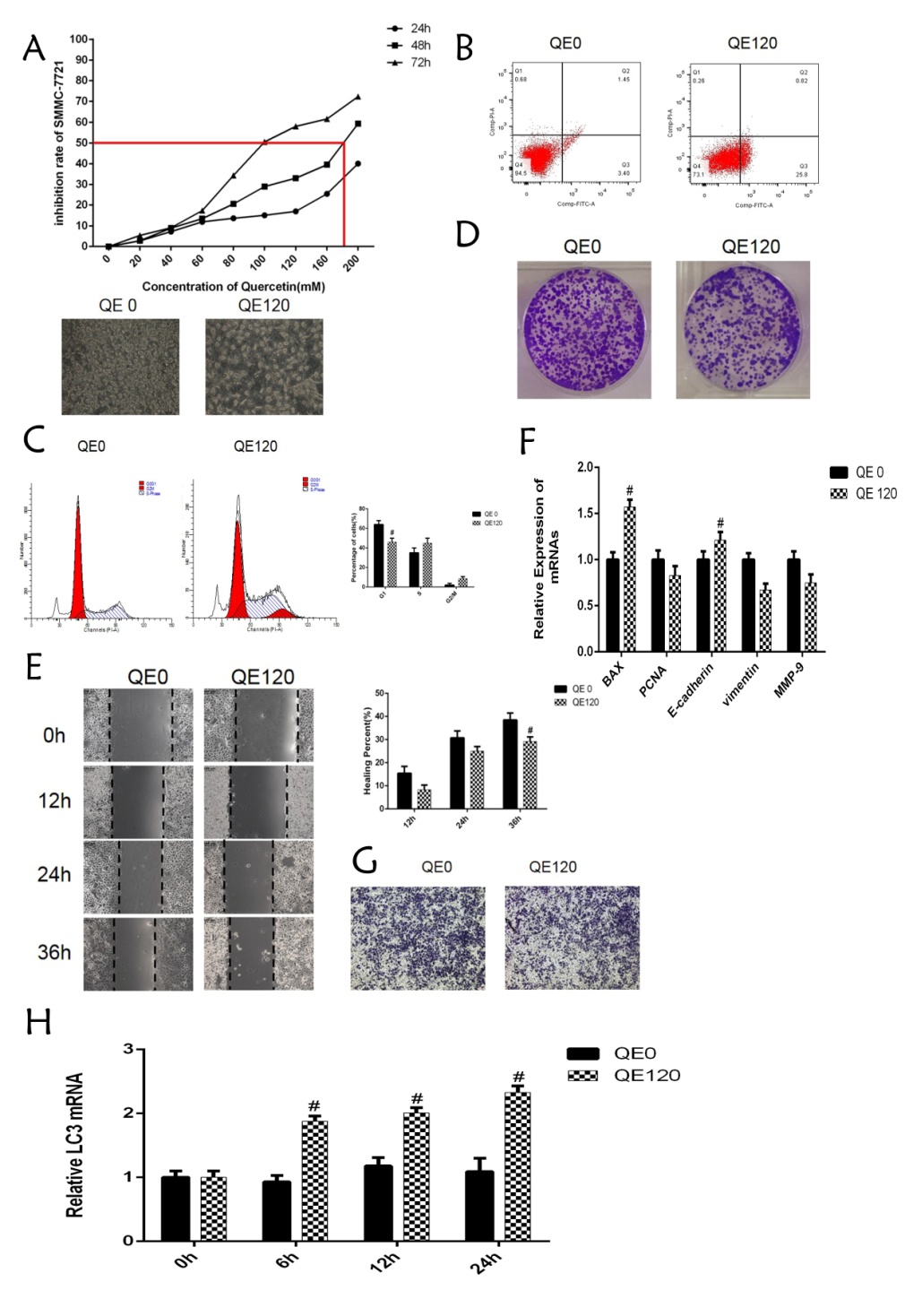
 (A) SMMC-7721 cells were treated with QE (0-200 μM) for 24,48 and 72 h. The CCK8 kit was used to monitor cell proliferation. And morphological changes in SMMC-7721 cells for 48h(magnification 400X).(B) Apoptosis of SMMC-7721 cells was determined by flow cytometry. (C) Cell cycle distribution of SMMC-7721 cells was determined by flow cytometry. The data are expressed as the mean±SD (^#^P<0.05 for QE120 versus QE0). (D)Colony formation of SMMC-7721. (E)The mRNA expression of Bax, PCNA , E-cadherin, Vimentin, and MMP9 were measured by qRT-PCR. The data are expressed as the mean±SD (^#^P<0.05 for QE120 versus QE0). (F)Wound healing assay for demonstrating the inhibitory effect of QE on the migration of L SMMC-7721 cells at 0,12,24 and 36 h following wounding. The data are expressed as the mean±SD (^#^P<0.05 for QE120 versus QE0). (G)Representative images of transwell invasion assays for the inhibitory effect of QE on the invasion ability of SMMC-7721 cells. (H)The mRNA expression of LC3 were measured by qRT-PCR. The data are expressed as the mean±SD (^#^P<0.05 for QE120 versus QE0).
